# Tail-scope: Using friends to estimate heavy tails of degree distributions in large-scale complex networks

**DOI:** 10.1038/srep09752

**Published:** 2015-05-11

**Authors:** Young-Ho Eom, Hang-Hyun Jo

**Affiliations:** 1 Laboratoire de Physique Théorique du CNRS, IRSAMC, Université de Toulouse, UPS, F-31062 Toulouse, France; 2 IMT Institute for Advanced Studies Lucca, Piazza San Francesco 19, Lucca 55100, Italy; 3 BK21plus Physics Division and Department of Physics, Pohang University of Science and Technology, Pohang 790-784, Republic of Korea; 4 BECS, Aalto University School of Science, P.O. Box 12200, FI-00076, Finland

## Abstract

Many complex networks in natural and social phenomena have often been characterized by heavy-tailed degree distributions. However, due to rapidly growing size of network data and concerns on privacy issues about using these data, it becomes more difficult to analyze complete data sets. Thus, it is crucial to devise effective and efficient estimation methods for heavy tails of degree distributions in large-scale networks only using local information of a small fraction of sampled nodes. Here we propose a *tail-scope* method based on local observational bias of the friendship paradox. We show that the tail-scope method outperforms the uniform node sampling for estimating heavy tails of degree distributions, while the opposite tendency is observed in the range of small degrees. In order to take advantages of both sampling methods, we devise the hybrid method that successfully recovers the whole range of degree distributions. Our tail-scope method shows how structural heterogeneities of large-scale complex networks can be used to effectively reveal the network structure only with limited local information.

Complex networks have served as a powerful mathematical framework to describe complex systems of nature, society, and technology 1,2,3,4,5. Most complex networks obtained from complex systems are known to be heterogeneous in various aspects 6,7,8,9. One of distinctive heterogeneous features in complex networks is the heavy-tailed degree distribution: A small number of highly connected nodes coexist with the large number of lowly connected nodes. Highly connected nodes or hubs found in heavy tails have significant roles on the evolution of complex networks and dynamics on such networks. For examples, the existence of hubs leads networks to endemic states in epidemic spreading 10,11, makes networks vulnerable to intended attacks 12, and contributes to the key functions of biological systems 13,14,15. Therefore, identifying the degree distribution and particularly hubs in the heavy tail of degree distribution is the essential step for the network analysis 16.

Owing to the rapid development of digital technologies, a huge amount of network data is being generated and recorded. In particular, the network data from social media like Twitter and Wikipedia contain tens of millions to billion nodes (users or articles). The role of social media on social dynamics such as public opinion formation, information diffusion, and popularity 17,18,19 is getting more crucial, requiring us to timely monitor the large-scale dynamics and to identify the network structure underlying these dynamics 4,20. However, since the social media are constantly growing and changing, the acquisition and analysis of complete network data is an extremely tricky task. Further, increasing public concerns on privacy issues about using these data can inhibit us from analyzing the complete network data 21.

Because of the above difficulties, degree distributions of complex networks need to be estimated based on partial information or by sampling nodes from networks. The simplest method could be to sample nodes randomly, which is called uniform node sampling (UNS). Since the number of nodes corresponding to the tail part of distribution is typically very small, those nodes are rarely sampled, limiting the *sampling resolution*. Accordingly, much larger statistical fluctuations are expected for the tail part of degree distribution estimated by UNS, when compared to its body part.

The friendship paradox (FP) 22,23,24 can shed light on how to effectively estimate the heavy tails of degree distributions. The FP states that the degree of an individual is on average smaller than the average degree of its friends or neighbors. The underlying mechanism behind the FP is the observational bias such that highly connected nodes are more likely to be observed by their neighbors. One can take advantage of this observational bias for the effective sampling of highly connected nodes. Indeed, the group made of friends of randomly chosen nodes turns out to contain highly connected nodes more than the group made of uniformly sampled nodes 24,25. Further, the FP has also been used for early detection of contagious outbreaks 21,26 and natural disaster 27, and for designing efficient immunization strategy 28. These are mainly based on the observation of activities of highly connected nodes via the FP rather than uniformly sampled nodes.

In this paper, we devise a novel sampling method, called tail-scope, to effectively estimate the heavy tails of degree distributions in large-scale complex networks. We exploit the observational bias of FP as a magnifying glass to observe heavy tails with better resolution and to overcome the resolution limit in the UNS. It is shown that the tail-scope method estimates heavy tails of empirical degree distributions in large-scale networks more accurately than the UNS. Finally, we suggest a hybrid sampling method taking advantages of both UNS and tail-scope methods to recover the whole range of degree distribution.

## Results

### Tail-scope: Estimating the tail of degree distribution using the friendship paradox

We consider a directed network *G* = *G*(*N*,*L*) with *N* nodes and *L* directed links. In case of undirected networks, each undirected link is considered as two directed links in both directions. For a node *i*, the in-degree *k*_*i*_ represents the number of incoming links to *i* from *i*'s in-neighbors, and the in-degree distribution is denoted by *P*(*k*). Similarly, one can define the out-degree as the number of out-neighbors.

Our goal is to effectively estimate the heavy tail of in-degree distribution, i.e., the region of *k* ≫ 1, by using partial information such as by sampling *n* nodes with *n* ≪ *N*. The observational bias of friendship paradox (FP) indicates that observation via friends can lead to the larger number of high degree nodes than that by the uniform node sampling (UNS), because the chance of a node being observed by its neighbors is proportional to the degree of the node. For this, we randomly choose *n* directed links and construct a set of nodes reached by following those links. The probability of finding a node of in-degree *k* in the set is proportional to 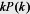 not to 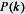, which we denote by 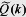:


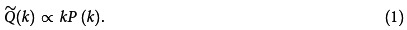


Then we obtain the estimated in-degree distribution as


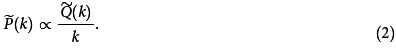


Thanks to the observational bias of FP, the estimated 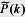 has the larger number of highly connected nodes and hence less statistical fluctuation for the tail part than when the UNS is used. Our method can be called *tail-scope*. Precisely, the sampling resolution characterized by the cutoff 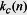 of the distribution is higher for the tail-scope method than for the UNS.

In order to demonstrate the effectiveness of tail-scope method for estimating the heavy tail of the distribution, we consider a network showing the power-law in-degree distribution with power-law exponent 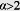 and minimum in-degree 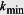:


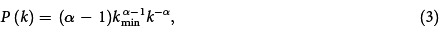


where we have assumed for convenience that the in-degree *k* is a continuous variable. At first, by randomly choosing *n* nodes (i.e., by UNS) we obtain the estimated in-degree distribution 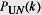 that is expected to be 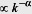. Due to the finiteness of *n*, we find the natural cutoff to the power-law tail as


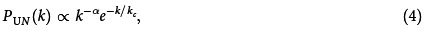


where *k*_c_ can be characterized by the condition


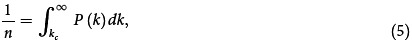


leading to


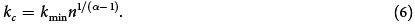


Next, for the tail-scope method, we expect from 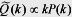 that


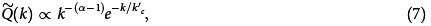



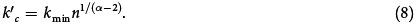


Then one gets the estimated in-degree distribution in Eq. (2):


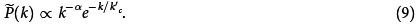


It is evident that the sampling resolution 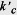 for the tail-scope case is higher than *k*_*c*_ for the UNS, precisely,


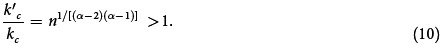


Therefore, our tail-scope method indeed outperforms the UNS for estimating the tail of the distribution. Since the tail-scope method is based on the uniform link sampling, it can also be called *link tail-scope*, mainly in order to distinguish from *node tail-scope* to be discussed in the next Subsection.

**Figure 1:**
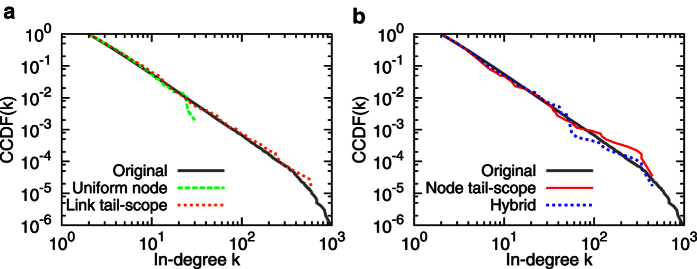
Comparison of in-degree distributions estimated by uniform node sampling, link tail-scope, node tail-scope, and hybrid methods to the original distribution for the Barabási-Albert scale-free network with 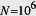 and minimum in-degree 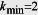. The sample size is *n* = 500. In all cases, complementary cumulative distribution functions (CCDFs) are presented.

We numerically test our calculations by constructing the Barabási-Albert (BA) scale-free network 6 with 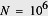, 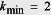, and 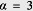, and then by sampling *n* = 500 nodes. From the calculations, we expect that 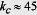 and 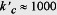, which are numerically confirmed as shown in Fig 1(A). In the figures, we have used the complementary cumulative distribution function (CCDF), defined as 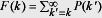, for clearer visualization.

### Node-based tail-scope method

Our tail-scope method is based on the uniform link sampling. However, in many realistic situations, we can use only the node-based sampling not the link-based sampling. For instance, most application programming interfaces (APIs) of social media like Twitter allow us to retrieve only the user-specific information rather than the relationship-based ones. Thus it is necessary to develop a sampling method using node-based data but aimed to simulate the link tail-scope method.

As social media APIs allow to get only user-specific local information in most cases, we assume that whenever a node is sampled or retrieved, we get the set of in- and out-neighbors for the sampled node. These constraints inevitably introduce correlations between sampled links, implying that any node-based tail-scope methods cannot be exactly mapped to the link tail-scope method. In addition, we assume that the number of retrievals, i.e., sampling size, is strictly limited to *n* for the fair comparison to other sampling methods, e.g., the UNS. We propose the node tail-scope method as follows.

#### *Node tail-scope method*:

- Step 1. Randomly choose *n*/2 nodes (called primary nodes) from the network and retrieve their out-neighbors to construct a set *A* of those out-neighbors.
- Step 2. Randomly choose *n*/2 nodes from the set *A* and retrieve their in-degrees to construct the distribution 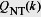.
- Step 3. Obtain the estimated in-degree distribution 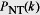 from 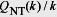.

Here the subscript NT of distributions is the abbreviation of node tail-scope. Note that as the total number of retrievals is limited to *n*, we use *n*/2 retrievals for getting out-neighbors, and the rest *n*/2 retrievals for getting in-degrees. However, there are more high degree nodes sampled than when the UNS is used, leading to the higher resolution for the tail-scope method. For a node sampled several times in Step 2, we consider each sampling as a different case.

By using the same BA network in the previous Subsection, we compare the performance of node tail-scope, shown in Fig. 1(B) to that of link tail-scope in Fig. 1(A). It is observed that there is no significant difference between two results.

### Performance of the node tail-scope method

In order to empirically compare the performance of node tail-scope method to the UNS, we consider several large-scale complex networks: four undirected networks and four directed networks. For details of these networks, see the Method Section and Table 1. From now on, we use the sample size *n* = 1000 in all cases. As mentioned, such small number of *n* is due to the practical constraint on the number of retrievals. When the constraint is relaxed, other sampling methods using graph traversal techniques (e.g., breadth first search) can be used, inducing more complicated observational biases 29.

**Figure 2:**
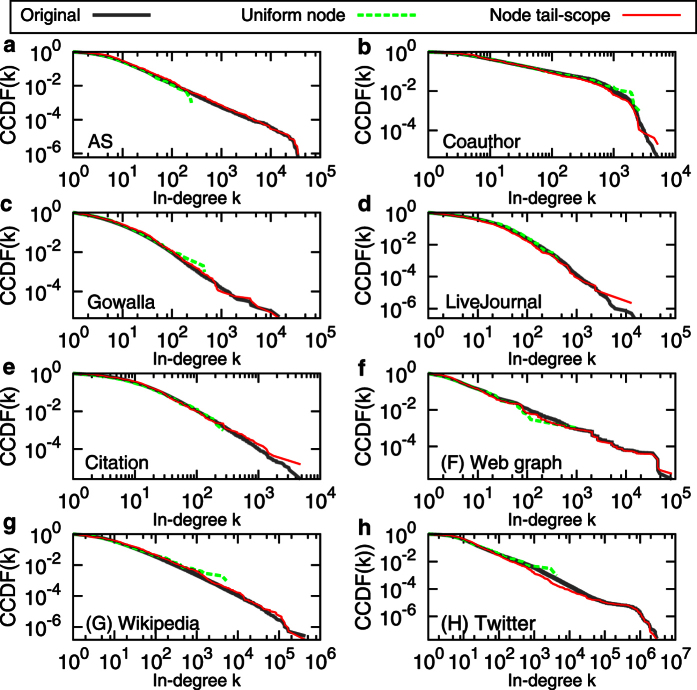
Comparison of in-degree distributions estimated by uniform node sampling and node tail-scope methods to the original distributions for several empirical directed and undirected networks. The sample size is *n* = 1000. In all cases, complementary cumulative distribution functions (CCDFs) are presented. For the details of the networks, see the Method Section and Table 1.

Figure 2 shows estimated in-degree distributions 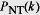 (node tail-scope) and 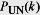 (UNS), in comparison to the original in-degree distribution 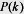 obtained from the complete set of nodes in the network. The agreements between original distributions and the distributions by node tail-scope method in the tail parts are remarkable, while some fluctuations are observed in the body parts. On the other hand, the distributions by the UNS show good agreements with the original distributions in the body parts, not in the tail parts. Note that the sample size *n* = 1000 is much smaller than the network size *N* ranging from hundreds of thousands to tens of millions nodes (see Table 1). We find that the results using *n* = 2000 and *n* = 4000 are qualitatively the same as the case of *n* = 1000.

For the quantitative comparison of performance by different sampling methods, we use Kolmogorov-Smirnov (KS) static *D*, defined as the maximum difference between two CCDFs. The KS *D*-static is mainly used as a part of KS test to reject null hypothesis. For example, it has been used to test if a given distribution has a power-law tail 16. In this paper, we simply use *D*-static to measure the agreement between the original in-degree distribution and the estimated in-degree distribution by each sampling method. The *D*-static for the node tail-scope method is obtained as


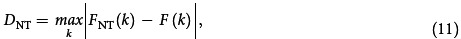


where 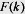 denotes the CCDF of the original in-degree distribution, and 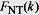 denotes the CCDF of 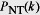. Similarly, 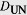 is defined for the UNS. The smaller *D*-static implies the better agreement to the original distribution.

Then, we define a *p*-value to compare the two considered sampling methods. The *p*-value represents the probability that the distribution by node tail-scope method has the smaller *D*-static with the original distribution than the distribution by the UNS, i.e.,


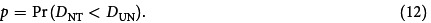


**Figure 3:**
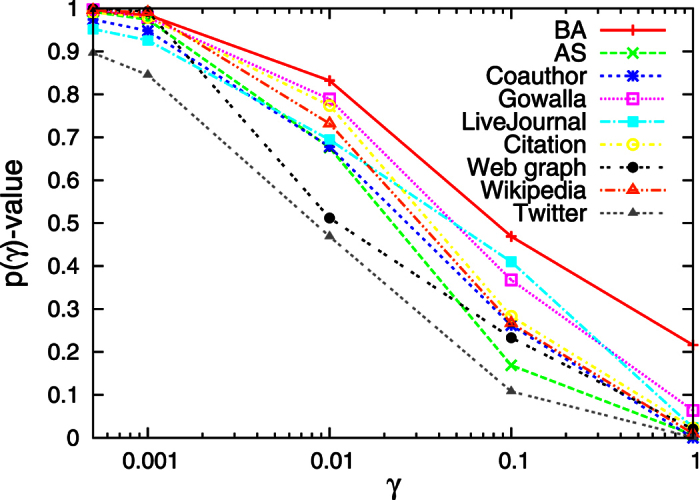
Performance of the node tail-scope method compared to the uniform node sampling for all the considered networks. 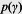 is calculated by Eq. (12) but with Kolmogorov-Smirnov *D*-statics defined only for the range of 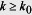, where 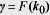. The smaller 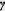 corresponds to the larger 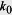. The larger 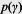-values imply the better performance of the node tail-scope method than the uniform node sampling. To get *p*-values, we used 1000 realizations of sampling, for each of which the sample size is *n* = 1000.

To focus on the tail part of the distribution, we compare the CCDFs only for the region of 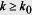, or equivalently for the fraction 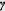 of high degree nodes, where 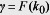. The case of 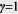 corresponds to the comparison for the entire range of in-degree. Figure 3 shows the values of 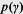 for different ranges of in-degree and for each considered network. It is found for all networks that the node tail-scope method clearly outperforms the UNS for the tail parts. The opposite tendency is observed when the entire range of the distribution is compared, because the UNS outperforms the node tail-scope for estimating the body part of the distribution. Since the sample size *n* is limited, the larger number of high degree nodes for the node tail-scope method results in the smaller number of low degree nodes and hence the larger fluctuations than the case of UNS.

As mentioned, since the node tail-scope method inevitably introduces correlations between sampled links, we now consider possible effects of degree correlations on the performance of node tail-scope method. As shown in Fig. 1, in the case of BA scale-free network with negligible degree correlation, the performance difference between the link tail-scope and the node tail-scope methods is not significant. We draw the same conclusion for considered empirical networks showing degree correlations, in terms of non-zero assortativity coefficients 8. For example, the assortativity coefficients are 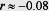 (AS), −0.029 (Gowalla), 0.467 (Coauthorship), and 0.045 (LiveJournal). These observations support the validity of our methods.

**Figure 4:**
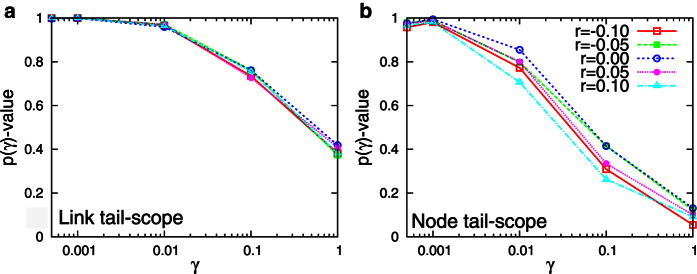
Performance of (A) link tail-scope method and of (B) node tail-scope method compared to the uniform node sampling for correlated scale-free networks with *N* = 50000 and degree exponent 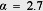 for 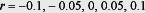, where *r* denotes the assortativity coefficient 8. 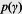 is calculated by Eq. (12) but with Kolmogorov-Smirnov *D*-statics defined only for the range of 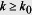, where 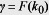. The smaller 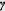 corresponds to the larger 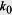. The larger 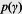-values imply the better performance of the link (node) tail-scope method than the uniform node sampling. To get *p*-values, we used 1000 realizations of sampling, for each of which the sample size is *n* = 500.

For making sure the validity of our methods for networks with non-zero degree correlation, we numerically consider correlated scale-free networks with tunable degree correlation used in 30. By using several scale-free networks with *N* = 5000, degree exponent 2.7 for 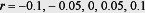, we obtain the *p*-values for each case. As expected, the link tail-scope method is barely influenced by the correlation (Fig. 4(A)). The node tail-scope method shows some effects of correlation but still gives us better sampling results than when UNS is used (Fig. 4 (B)). Overall, the sampling results can be affected if the degree correlation is quite strong. However, our method still performs better for sampling the tail parts than the UNS.

### Hybrid method for recovering the whole distribution

It is evident that the UNS and the node tail-scope method are good at sampling low and high degree nodes, respectively. In order to take advantages of both methods, we suggest the *hybrid method* for recovering the whole range of the distribution. It is notable that at Step 1 in our node tail-scope method, *n*/2 primary nodes are randomly chosen and hence their in-degrees can be utilized for the low degree region. From the primary nodes, we get the in-degree distribution 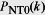. Then the hybrid distribution is obtained by


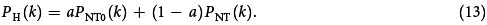


The weight parameter 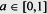 can be chosen according to which part of the distribution is focused. Here we set as 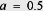.

**Figure 5:**
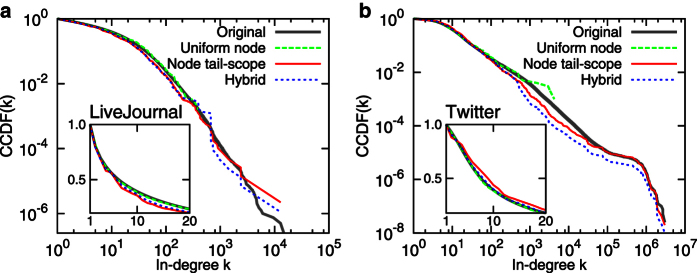
Comparison of in-degree distributions estimated by uniform node sampling, node tail-scope, and hybrid methods to the original distributions for networks of LiveJournal (**A**) and Twitter (**B**). The insets show results for the range of 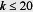. The sample size is *n* = 1000. In all cases, complementary cumulative distribution functions (CCDFs) are presented.

**Figure 6:**
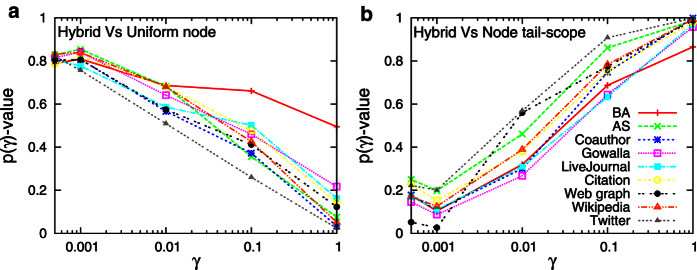
Performance of the hybrid method compared to the uniform node sampling (A) and to the node tail-scope method (B) for all the considered networks. 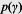 is calculated by Eq. (12) but with Kolmogorov-Smirnov *D*-statics defined only for the range of 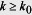, where 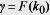. The smaller 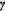 corresponds to the larger 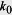. The larger 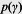-values imply the better performance of the hybrid method than the uniform node sampling (**A**) or the node tail-scope method (**B**). To get *p*-values, we used 1000 realizations of sampling, for each of which the sample size is *n* = 1000.

The hybrid method performs well for the BA network in Fig. 1(B) as well as for empirical networks, two of which are shown in Fig. 5. As expected, the distributions estimated by the hybrid method fit the original distributions better than the UNS for the tail parts, and better than the node tail-scope method for the body parts (see insets in Fig. 5). These findings are also consistent with the values of 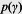 shown in Fig. 6: The larger values of 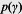 for small values of 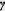 in Fig. 6(A) imply the better performance of the hybrid method than the UNS for the tail parts. The larger values of 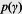 for large values of 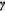 in Fig. 6(B) imply the better performance of the hybrid method than the node tail-scope for the body parts. Therefore, we conclude that the hybrid method successfully recovers the whole range of in-degree distributions, by taking advantages of both the UNS and the node tail-scope methods. Other values of *a* = 0.25 and *a* = 0.75 have been also tested and all results are as expected.

## Discussion

Modern societies have been shaped by large-scale networked systems like World Wide Web, social media, and transportation systems. Monitoring global activities and identifying the network structure of these systems are of utmost importance in better understanding collective social dynamics. However, increasing size of data from these systems and growing concerns on privacy issues about using these data make the exhausted analysis of complete data sets infeasible. Thus, effective and efficient estimation of large-scale networks based on the small sample size or partial information is necessary. One of the simplest method could be uniform node sampling (UNS). The UNS has drawbacks in particular for estimating the heavy tails of degree distributions, due to the limited sampling resolution and large statistical fluctuations. Since high degree nodes found in the heavy tails are in many cases very important to characterize the structure and dynamics of complex networks, we propose the tail-scope method, which is the effective and efficient sampling method for estimation of heavy tails of degree distributions.

Provided that the sample size is limited, it is inevitable that the larger number of high degree nodes by the tail-scope method leads to the smaller number of low degree nodes than when the UNS is used. In order to take advantages of both the tail-scope and the UNS, we propose the hybrid method to recover the whole range of degree distributions. In this paper, we have considered a very simple form of hybrid method by superposing the estimated degree distributions of the UNS and the tail-scope. It turns out that the hybrid method performs better than the UNS for the tail parts, and better than the tail-scope for the body parts. Devising more general and better hybrid methods will be interesting as a future work, e.g., one can use the degree-dependent weight parameter *a* in Eq. (13).

Our tail-scope method can be also used for estimating high attribute nodes found in the heavy tail of attribute distribution. The attribute of a node can be its activity, income, happiness, and so on. Recently, the generalized friendship paradox (GFP) has been observed and analyzed in complex networks 24,30. The GFP states that the attribute of a node is on average lower than the average attribute of its neighbors. In the network showing the positive correlation between degrees and attributes, high degree nodes tend to have higher attributes. It implies that the high attribute nodes are more likely to be observed by their neighbors. Such generalized observational bias can be exploited to effectively estimate high attribute nodes who play important roles, e.g., in early detection of new trends or in designing efficient immunization strategies. Thus, it would be very interesting to generalize our tail-scope method to other attributes of nodes, especially for the large-scale complex networks.

Our tail-scope method shows how structural heterogeneities can help us reveal the network structure only with limited information. By exploiting such heterogeneities of complex networks we can properly evaluate priority and importance of each node in the networks. It is getting more important to better understand the heterogeneities since they are key features characterizing the complexity of large-scale networks.

## Methods

### Data description

In this paper, we consider eight empirical networks: four of them are undirected and the others are directed. The summary of the networks is presented in Table 1. The detailed feature of each network is as following.

*AS.* We used an Autonomous Systems (ASs) data set on Internet topology graph constructed in (ref. 31). The nodes are autonomous systems and the links are formed where two ASs exchange traffic flows. The size of network is *N* = 1696415.

#### Coauthorship

We used a coauthorship network constructed in (ref. 24). The nodes are scientists and the links are formed whenever two scientists coauthored the paper. The network size is *N* = 242592.

#### Gowalla

We used a Gowalla friendship network constructed in (ref. 32). Gowalla is a location-based social networking service. Each user defines a node. The network size is *N* = 196562.

#### LiveJournal

We used a LiveJournal friendship network constructed in (ref. 33). Livejournal.com is a social networking service for blog, journal, and diary. The nodes are users of LiveJournal and the users can declare friendship to another user, defining a link. The network size is *N* = 3997962.

#### Citation

We used a citation network constructed in (ref. 34). The network is based on the bibliographic database from 1893 to 2009 provided by American Physical Society (APS). The nodes are articles published in APS journal such as Physical Review Letters or Physical Review E and the directed links represent the citation relation between articles. The network size is *N* = 463349.

#### Web graph

We used a web graph constructed in (ref. 35). The nodes represent webpages in the domains of berkely.edu and stanford.edu domains, and the links are hyperlink between webpages. The network size is *N* = 685230.

#### Wikipedia

We used an English Wikipedia network constructed in (ref. 36). The Wikipedia data set was collected in February 2013. The nodes are English Wikipedia articles and the links are hyperlinks between those articles. The network size is *N* = 4212493.

#### Twitter

We used a Twitter users network constructed in (ref. 37). The nodes are Twitter users and the links between users represent the following relations in Twitter. The network size is *N* = 41652230.

## Additional Information

**How to cite this article**: Eom, Y.-H. and Jo, H.-H. Tail-scope: Using friends to estimate heavy tails of degree distributions in large-scale complex networks. *Sci. Rep.* **5**, 9752; doi: 10.1038/srep09752 (2015).

## Figures and Tables

**Table 1:** Basic statistics of empirical undirected and directed networks.

**Undirected network**	***N***	**〈*****k*****〉**	**Directed network**	***N***	**〈*****k*****〉**
AS	1696415	13.1	Citation	463349	12.2
Coauthorship	242592	59.6	Web graph	685230	12.3
Gowalla	196562	9.7	Wikipedia	4212493	26.4
LiveJournal	3997962	17.3	Twitter	41652230	36.6

*N* denotes the total number of nodes and **〈***k***〉** denotes the average in-degree. The isolated nodes have been excluded for the analysis.
